# Electrode Design for MnO_2_-Based Aqueous Electrochemical Capacitors: Influence of Porosity and Mass Loading

**DOI:** 10.3390/ma14112990

**Published:** 2021-06-01

**Authors:** Camille Douard, Laurence Athouël, David Brown, Olivier Crosnier, Guillaume Rebmann, Oliver Schilling, Thierry Brousse

**Affiliations:** 1 IMN, Institut des Matériaux Jean Rouxel, Université de Nantes, CNRS, F-44000 Nantes, France; camille.douard@cnrs-imn.fr (C.D.); david.brown@cnrs-imn.fr (D.B.); olivier.crosnier@univ-nantes.fr (O.C.); 2 RS2E, Réseau sur le Stockage Electrochimique de l’Energie, Research Center on Batteries and Supercapacitors, CNRS FR 3459, 33 rue Saint Leu, CEDEX, 80039 Amiens, France; 3 Innovation Center, Prince Minerals SRL, Rue du Bois, B-7334 Saint-Ghislain, Belgium; grebmann@princecorp.com; 4 Innovation Center, Prince Specialty Products LLC, 610 Pittman Road, Baltimore, MD 21236, USA; oschilling@princecorp.com

**Keywords:** MnO_2_, supercapacitor, electrode fabrication, mass loading, pseudocapacitance

## Abstract

The purpose of this study is to highlight the influence of some fabrication parameters, such as mass loading and porosity, which are not really elucidated and standardized during the realization of electrodes for supercapacitors, especially when using metal oxides as electrode materials. Electrode calendering, as one stage during the fabrication of electrodes, was carried out step-by-step on manganese dioxide electrodes to study the decreasing porosity effect on the electrochemical performance of a MnO_2_ symmetric device. One other crucial parameter, the mass loading, which has to be understood and well used for realistic supercapacitors, was investigated concurrently. Gravimetric, areal and volumetric capacitances are highlighted, varying the porosity for low-, medium- and large-mass loading. Low-loading leads to the best specific capacitances but is not credible for realistic supercapacitors, except for microdevices. Down 50% porosities after calendering, capacitances are increased and become stable faster, suggesting a faster wettability of the dense electrodes by the electrolyte, especially for high-mass loading. EIS experiments performed on electrodes without and with calendering lead to a significant decrease of the device’s time response, especially at high loading. A high-mass loading device seems to work as a power battery, whereas electrode calendaring, which allows decreasing the time response, leads to an electrical behavior closer to that expected for a supercapacitor.

## 1. Introduction

The tremendous efforts dedicated to the search of new materials for electrochemical capacitors, so-called supercapacitors, should not hide the practical difficulties for reporting the performance of the related electrodes. Indeed, fabrication parameters are crucial steps that should in principle exacerbate the performance of the electrode materials. However, for some obscure reasons, only a few papers are dedicated to electrode fabrication [1,2,3,4,5], and even fewer focus on the standardization of the electrode preparation and testing [6]. Prototyping ‘real life’ cells is also seldom reported [7,8]. Most of these studies are related to the use of carbon-based electrodes [9,10]. Unfortunately, this seems to be a common feature with the electrode battery topic, unlike other communities which have clearly defined their testing protocols, such as for photovoltaic materials [11,12,13,14]. Indeed, in the field of batteries and supercapacitors, authors are reporting gravimetric values for the capacity, capacitance and energy and power densities, with electrode loadings ranging from a few µg·cm^−2^ (µg of active material per footprint area of the related electrode) to several tens of mg·cm^−2^. This was the starting point for several extravagant announcements concerning materials with properties that were claimed as miraculous but were in fact obtained with extremely limited amounts of material in the electrodes. Some authors have tried to emphasize the fact that it was not correct to extrapolate low-mass loadings to device performance [15,16,17,18] but only a few groups around the world caught the message [19]. As an exception to this assessment, a recent study pointed out the effect of MnO_2_ mass loading on capacitive deionization devices and showed that it was a key parameter in this application as well [3]. Nevertheless, the aim of supercapacitor and battery manufacturing companies is to lower the mass and volume of all the cell components in order to increase the amount of active material inside their devices, thus optimizing the related energy and power densities. Indeed, this implies loading a realistic mass of active materials in the electrodes.

Mass loading is not a unique issue to electrodes for batteries and supercapacitors. The electrode porosity also plays a major role in the design of devices: a high porosity (as prepared electrodes often exhibit porosity up to 80 vol.%) seems to be efficient to enable the ions (and solvent molecules) to freely access the surface of the electrodes. Nevertheless, a high porosity leads to a low volumetric capacity/capacitance due to the confinement of a small amount of active material in a huge volume. Moreover, a large porosity hinders electronic conductivity, which must also be taken into account together with ionic conductivity [10,20]. However, preparing a very dense electrode is also counterproductive in the sense that it severely limits the electrode/electrolyte interaction and leads to an inaccessible area of active material to the ions of the electrolyte. This effect in turn drastically limits the volumetric capacity of the electrode. Subsequently, a trade-off between a reasonable porosity and a high volumetric capacity/capacitance has to be found. This is also a major concern for battery manufacturing [21]. In industrial processes, a calendering step is most often performed in order to decrease porosity while keeping reasonable access of ions from the electrolyte to a significant part of the electrode surface. The action of calendering is the process by which a composite electrode deposited onto its current collector is laminated through two metal cylinders in a continuous way to a certain thickness. As a result, the mass loading is the same as for the as-coated electrode while the volumetric loading (in mg·cm^−3^) is significantly improved. Such a process has been the topic of many modeling and experimental studies in the field of lithium-ion batteries [22,23,24,25,26,27], organic lithium-ion batteries [28], lithium-sulfur batteries [29] or zinc-based batteries [30]. It is most rarely reported for supercapacitors [31].

It is obviously not possible to apply such industrial processes to materials resulting from synthesis routes that can only provide few milligrams of powder, or even less. This should not be a problem as long as the authors reporting on such materials are providing the mass loading of the related electrodes and they realize that the use of a few hundred µg of materials per cm^2^ is definitely preventing the use of gravimetric values. In such a situation, only areal values matter, as is the case in the field of microdevices (micro-batteries and micro-supercapacitors) or flexible/implantable devices. Electrode fabrication is a means to an end and not an end in itself. However, authors must realize that every reported performance is usually translated by readers into device performance. Thus, providing unrealistic values is often misleading for stakeholders, either academics or industrial teams.

In this study, we will try to demonstrate the effect of mass loading and other key parameters, namely electrode porosity and wettability, on the performance of MnO_2_-based symmetrical devices. Commercially available MnO_2_ powder was used for this study, which allowed the preparation of ‘real life’ electrodes with mass loading compatible with industrial processes. We hope this will trigger more practical work to improve our knowledge of how and to which extent process parameters influence the energy and power densities of the related devices.

## 2. Materials and Methods

### 2.1. Materials

All purchased chemical products, as polyvinylidene fluoride PVDF (Solef^®^ 5130, Solvay, Bollate, Italia), N-methyl-pyrrolidinone NMP, 99% extra pure (Acros Organics^TM^, Geel, Belgium), lithium nitrate anhydrous LiNO_3_, extra pure (Fisher Chemicals, Illkirch, France) were used as received. Commercially available α-MnO_2_ cryptomelane-type manganese dioxide, MnO_2_ H.S.A. (high surface area), was supplied by Prince Corporation (Baltimore, MD, USA). MnO_2_ composite electrodes were elaborated using commercial carbon black additives, a partially graphitized nano-sized one (Pureblack^®^ grade 205-110 carbon from Superior Graphite Co. Chicago, IL, USA, 45 m^2^/g), named SG in the present study, or a very high purity one (C-Nergy™ Super C45 carbon black from Imerys Graphite & Carbon Co., Willebroek, Belgium, 45 m^2^/g), named C45. All products were used without further treatments. The PVDF powder was dissolved in NMP (7 wt.% of PVDF) to be used for the composite inked electrodes. All electrochemical measurements were carried out using neutral aqueous 5M LiNO_3_ electrolyte adjusted to pH 7.

### 2.2. Preparation of the Electrodes and Symmetric Devices

**Figure 1:**
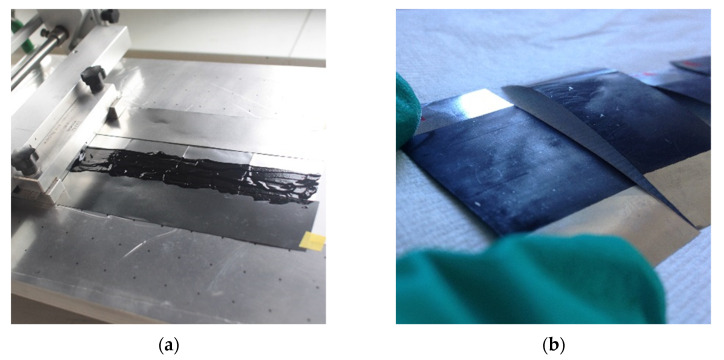

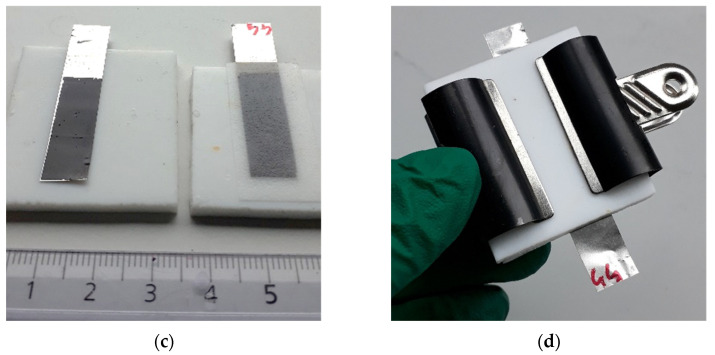
Fabrication steps of α-MnO_2_ ink electrodes and the symmetric device: (**a**) Ink coating using a doctor blade apparatus; ink containing a dry matter of c.a. 25 wt.% is coated on stainless steel current collectors; the gap between the doctor blade and the substrate can be adjusted in order to prepare MnO_2_-based electrodes with different mass loadings; (**b**) After drying at 60 °C for 1 night under vacuum for 1–2 h, the coated stainless steel substrate is cut into rectangular-shaped electrodes; (**c**) Electrodes are assembled according to a face-to-face two-electrode design; the two electrodes are separated by a cellulosic separator impregnated with 5M LiNO_3_ neutral aqueous electrolyte and the stack is pressed between two Teflon^®^ plates; (**d**) α-MnO_2_ symmetric two-electrode cell; the metallic clamps enable the application of the same pressure to all the symmetrical devices.

The electrodes were prepared from an ink with a weight % composition of 75/15/10 for MnO_2_ as active material, SG or C45 carbon black as the conductive additive part and PVDF (7 wt.%, dissolved in NMP) as the binder, respectively. During the inking process, small amounts of NMP were added and adjusted to systematically obtain a quantity of dry matter of c.a. 25 wt.% in the ink. A tape-casting method (doctor blade coating apparatus, Braive Instrument, Liège, Belgium) was used to spread the ink on an AISI 316 stainless steel foil substrate (from Goodfellow, Cambridge, UK, 25 μm thick). About 5 pieces of stainless steel (6 cm in length × 2 cm in width) have been assembled side-by-side by the back face before coating a strip of ink at the center of the five electrodes (Figure 1a). The gap between the doctor blade and the substrate was adjusted at three different wet tape thicknesses, 300 μm, 450 μm and 650 μm, in order to obtain three different active material (AM) loadings for the electrodes, respectively: a low (2–3 mg_AM_·cm^−2^), a medium (5–6 mg_AM_·cm^−2^) and a large one (10–12 mg_AM_·cm^−2^) (Figure 1b). Then, the film tape was dried at 60 °C for 1 night under vacuum for 1–2 h. Rectangular-shaped electrodes, between 2 and 3.5 cm long and 2 cm wide, were then obtained after cutting from the dry film tape. The dry film thicknesses reach 50–60 μm, 90–110 μm and 170–200 μm for low, medium and large loadings, respectively, leading to calculated porosities of 70–77%. It can be noted that the porosity of the as-prepared electrodes does not depend on the mass loading.

In order to study the effect of electrode porosity on the electrochemical behavior and to increase the active material density of the electrode, heat calendering was performed at 60 °C using a pressure-controlled hot rolling machine (4” Width Electric Hot Rolling Press with Variable Speed MSK-HRP-01 from MIT Co. KJ Group Richmond, CA, USA). From the upper thickness, the roller gap was decreased by 10 μm step after 5 successive calenderings at a rolling speed of 14 mm·s^−1^ until an adjusted final gap obtained the required thicknesses. The final pressed film thicknesses reached around 30–50 μm, 50–80 μm and 80–130 μm for low, medium and large loadings, respectively, leading to porosities ranging from 41% to 73% depending on the active material loading, the nature of the carbon black additive and the roller gap.

Symmetric devices were assembled according to a two-electrode configuration using inked electrodes, noted ‘electrodes’ in the following paragraphs. Each final coated piece was cut in two parts to obtain two similar electrodes (~2 cm^2^) intended for the face-to-face two-electrode device (Figure 1c). The two-electrode cells were assembled with a cellulosic separator (Whatman® Grade 41 ashless quantitative filter paper, VWR International, Fontenay-sous-Bois, France, 215 μm thick), and the stack was pressed between two Teflon^®^ plates. Two metallic clamps help to maintain the pressure of the stack as seen in Figure 1d. The electrodes and the separator were previously soaked in the 5M LiNO_3_ neutral aqueous electrolyte. The volume of electrolyte was estimated to be 260 μL·cm^−2^ (~0.3 g·cm^−2^) per cm^2^ of device. It must be noted that this volume of electrolyte contains more than 10 times the maximum amount of ions necessary to charge the electrodes, thus avoiding ion depletion from the electrolyte upon cycling the cells. The cells were intentionally used just after impregnation to evidence how many cycles are necessary to reach a steady state depending upon the mass loading and porosity.

### 2.3. Material Characterizations and Electrochemical Measurements

Structural and morphological characterizations of cryptomelane-type α-MnO_2_ powder were investigated by XRD using a PANalytical X’Pert Pro X-ray diffractometer (Palaiseau, France) with an X’Celerator detector and Cu Kα radiation (0.15406 nm), and by SEM using a Zeiss Merlin^TM^ FE-SEM field emission scanning electron microscope (Jena, Germany). Volume measurements and density calculations were performed using a Micromeritics AccuPyc^TM^ II 1340 gas displacement pycnometry system (Norcross, GA, USA) with helium inert gas and the specific surface area using the BET method. Electrochemical investigations were conducted in 5M LiNO_3_ neutral aqueous electrolyte on a Biologic VMP3 galvanostat-potentiostat (Grenoble, France) operated under EC-Lab^®^ software v11.33.

The composite MnO_2_ electrode as the working electrode was electrochemically characterized by cyclic voltammetry and long-time cycling on 150 cycles in a three-electrode cell using Ag/AgCl as the reference electrode (+0.196 V vs. NHE reference at 25 °C) and a platinum gauze as the counter electrode. Electrochemical measurements were carried out between 0 V and 1 V versus Ag/AgCl and specific capacitances C (F·g^−1^) determined for 20 mV·s^−1^, 10 mV·s^−1^ and 5 mV·s^−1^ cycling rates by integrating the reductive part to obtain the discharge charge Q (C) and dividing the charge by the mass of active material m (g) and the width of the potential window ΔE (V), i.e.:

C = Q/(ΔE × m)(1)

All specific capacitances are given per grams of MnO_2_ active material.

In the same way, composite electrodes were characterized in the two-electrode assembly by cyclic voltammetry using the same previous experimental conditions. Alternatively, the devices were cycled by constant current galvanostatic charge/discharge (CCGCD) with current densities ranging from 0.1 to 10 A·g^−1^. For clarity purpose, the specific capacitances (F·g^−1^) and areal capacitances (F·cm^−2^) reported in this study were determined at 20 mV·s^−1^ and/or 10 mV·s^−1^ for different electrode porosities, the three different loadings, low (2–3 mg·cm^−2^), medium (5–6 mg·cm^−2^) and large (10–12 mg·cm^−2^) and for SG and/or C45 carbon blacks, after 50 cycles and 300 cycles. Quite similar values were measured with CCGCD. Long-term cycling for 10 h was performed on 300 cycles for 50% and 70% porosities with C45 carbon black and each loading followed the impedance changes and the Nyquist diagram by potential electrochemical impedance spectroscopy (PEIS), before cycling, after every 50 cycles at 20 mV·s^−1^, 10 cycles at 10 mV·s^−1^ and after 300 cycles. EIS analytical conditions were a frequency range from 200 kHz down to 2 mHZ at the open-circuit voltage E_OC_ for a potential amplitude of 20 mV. The response time as a function of the mass loading was highlighted for both 50% and 70% porosities from the Bode plots of the C’ real and C’’ imaginary capacitance contributions vs. the frequency according to EIS data treatment proposed by Taberna et al. [32].

## 3. Results and Discussion

### 3.1. MnO_2_ Structural Characterizations

**Figure 2:**
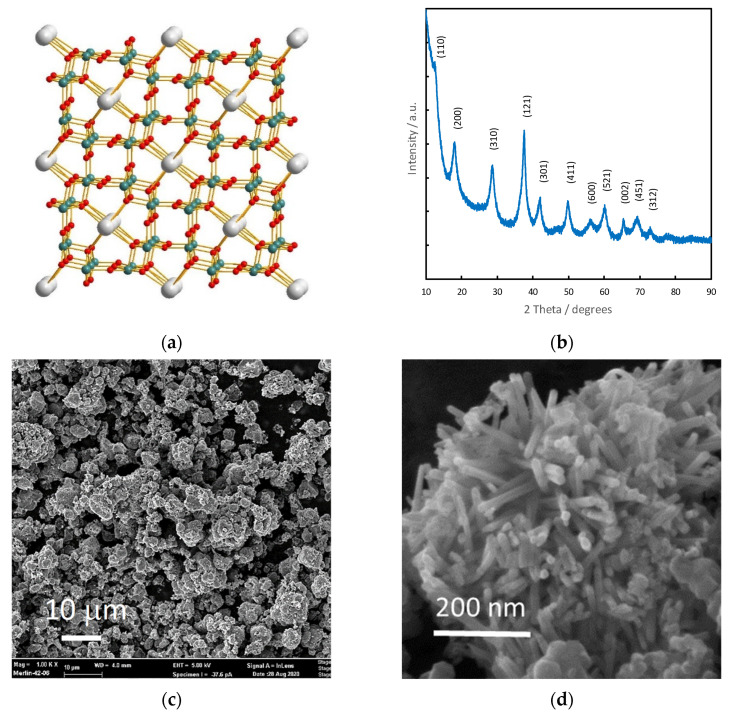
Structural and morphological characterization of cryptomelane α-MnO_2_: (**a**) Crystallographic structure; (**b**) XRD pattern; (**c**) SEM micrograph (scale bar 10 µm); (**d**) SEM micrograph (scale bar 200 nm).

Synthetic cryptomelane is a mixed-valence compound K_x_Mn^4+^_(8−x)_Mn^3+^_x_O_16_ (x = 1.33) with a 2 × 2 tunneling MnO_6_ octahedra building blocks framework (4.6 Å × 4.6 Å size) [33,34,35] as depicted schematically in Figure 2a. The as-received commercial powder is a well-crystallized manganese dioxide in compliance with the XRD pattern shown in Figure 2b. X-ray diffraction peaks and (hkl) indexations are consistent with the pattern of cryptomelane (JCPDS 29-1020) and crystallographic data from the literature [36] expecting a tetragonal symmetry with an I4/m space group and lattice parameters a = 9.866 Å and c = 2.872 Å [37,38]. Accordingly, the cell volume is 279.555 Å^3^, leading to a crystal density of 4.441 g·cm^−3^ for cryptomelane.

### 3.2. Porosity Determination of α-MnO_2_

The morphology of the α-MnO_2_ powder can be observed in Figure 2c,d which shows aggregates of well-distributed grains with an average diameter of 5 μm (Figure 2c). Higher magnification reveals nanorods-like microstructure (Figure 2d). The nanorods are about 100 nm in length and less than 10 nm in diameter. The BET surface area measured using nitrogen adsorption isotherms is close to 208 m^2^·g^−1^. The geometric surface of the nanorods can be calculated as follows:

S = 4/(d × ρ)(2)

with d representing the mean diameter of the nanorods and ρ the theoretical density. The calculation gives a diameter value of ~5 nm which seems consistent with the SEM images (Figure 2d). Thus, the α-MnO_2_ powder is made of 5 µm diameter aggregates of dense nanorods 5 nm in diameter and 100 nm in length. The N_2_ adsorption isotherm, not shown here, exhibits an IV-type isotherm from the IUPAC classification, suggesting the presence of mesopores.

The helium pycnometry measurement leads to an effective density of 3.8 g·cm^−3^, roughly conclusive to a probably micro or mesoporosity of 15% probably formed upon the piling-up of the nanorods. At a larger macroscopic scale, tap density measurements were performed on various volumes of cryptomelane powder, resulting in an average tap density of 0.82 g·cm^−3^ for α-MnO_2_ in 1 cm^3^ of a geometric volume, which is consistent with previously reported values [39]. This value corresponds to ~81.5% porosity in the tap powder.

### 3.3. Electrochemical Characterization of a Single α-MnO_2_ Electrode in a 3-Electrode cell

**Figure 3:**
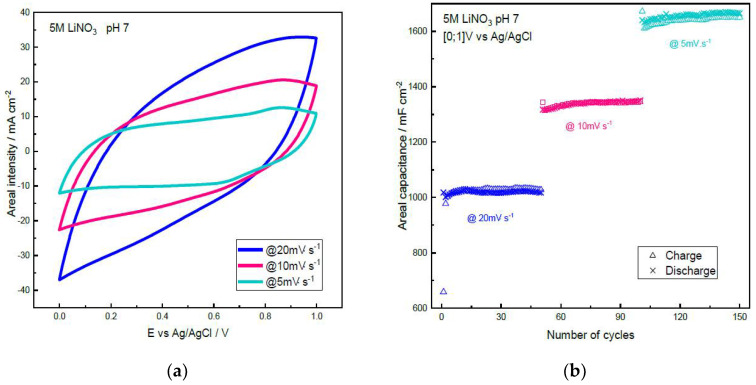
Three-electrode cell cyclic voltammetry of cryptomelane α-MnO_2_ in 5M LiNO_3_ between 0 V and 1 V vs. Ag/AgCl: (**a**) CV for 20 mV·s^−1^, 10 mV·s^−1^ and 5 mV·s^−1^; (**b**) Areal capacitance evolution at various cycling rates on 150 cycles.

Cryptomelane α-MnO_2_ was investigated by cyclic voltammetry using a three-electrode cell in 5M LiNO_3_ neutral aqueous electrolyte (α-MnO_2_ mass ~12 mg·cm^−2^, porosity ~75%). Figure 3a shows the cyclic voltammograms obtained between 0 V and 1 V vs. Ag/AgCl at 20 mV·s^−1^, 10 mV·s^−1^ and 5 mV·s^−1^. A typical rectangular shape relative to a pseudocapacitive storage mechanism [40,41] is observed, showing an increasing resistive behavior of the electrode as the scan rate increases. The specific capacitances were determined from the CVs, leading to typical values for cryptomelane of 86 F·g^−1^ at 20 mV·s^−1^, 113 F·g^−1^ at 10 mV·s^−1^ and 139 F·g^−1^ (per g of active material) at 5 mV·s^−1^ [42]. These values compare well with composite electrodes containing manganese dioxide and 0–20 wt% carbon black with a high total mass loading of 55 mg·cm^−2^, which exhibited a specific capacitance (SC) of 128 F·g^−1^ (g of MnO_2_) at a scan rate of 2 mV·s^−1^ in 0.5M Na_2_SO_4_ solutions [19].

Stable long-term cycling of the capacitance on 150 cycles is reported in Figure 3b, showing the evolution of the areal capacitance by cm^2^ of the electrode for the three scan rates (1.02 F·cm^−2^, 1.35 F·cm^−2^ and 1.66 F·cm^−2^ for, respectively, 20, 10 and 5 mV·s^−1^). These values translate into 0.77 F·cm^−2^, 1.01 F·cm^−2^ and 1.25 F·cm^−2^ when taking into account the total mass of the electrode. These values compare well with that of an activated carbon electrode in a standard commercial device which is close to 1 F·cm^−2^ (~100 F·g^−1^ for the activated carbon electrode and ~10 mg·cm^−2^ mass loading) [43]. It is to be noted that the capacitances are equivalent during the charge and discharge (Figure 3b), revealing good reversibility of the charge storage.

Subsequently, when assembling a symmetrical cell with two α-MnO_2_ electrodes loaded at ~12 mg·cm^−2^, the maximum capacitance of the device should be 22 F·g^−1^ at 20 mV·s^−1^ and 28 F·g^−1^ at 10 mV·s^−1^ (per g of total active material in the device).

### 3.4. Electrochemical Characterization of the Symmetric Devices

**Figure 4:**
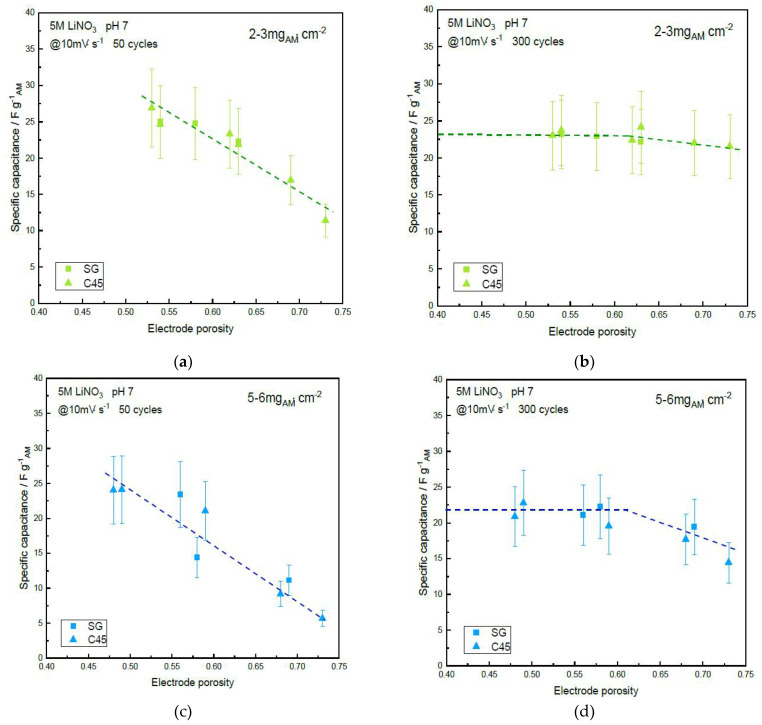

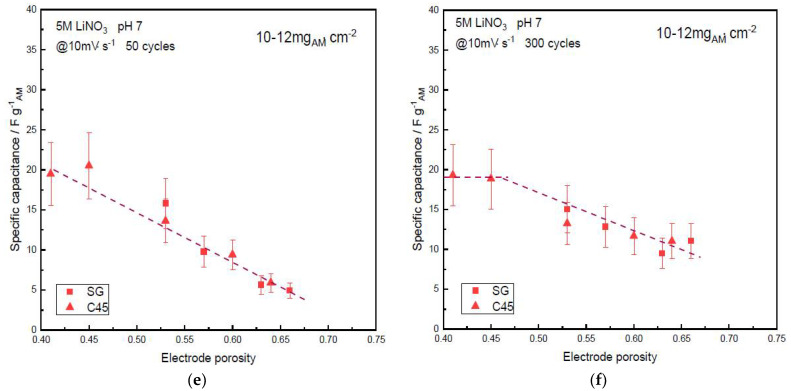
Specific capacitance vs. electrode porosity of the α-MnO_2_ symmetric supercapacitor device using SG or C45 carbon black additive, after cycling in 5M LiNO_3_ at 10 mV·s^−1^: (**a**) Low-mass loading, 50 cycles; (**b**) Low-mass loading, 300 cycles; (**c**) Medium-mass loading, 50 cycles; (**d**) Medium-mass loading, 300 cycles; (**e**) Large-mass loading, 50 cycles; (**f**) Large-mass loading, 300 cycles. Dashed lines are guidelines for the eyes.

**Figure 5:**
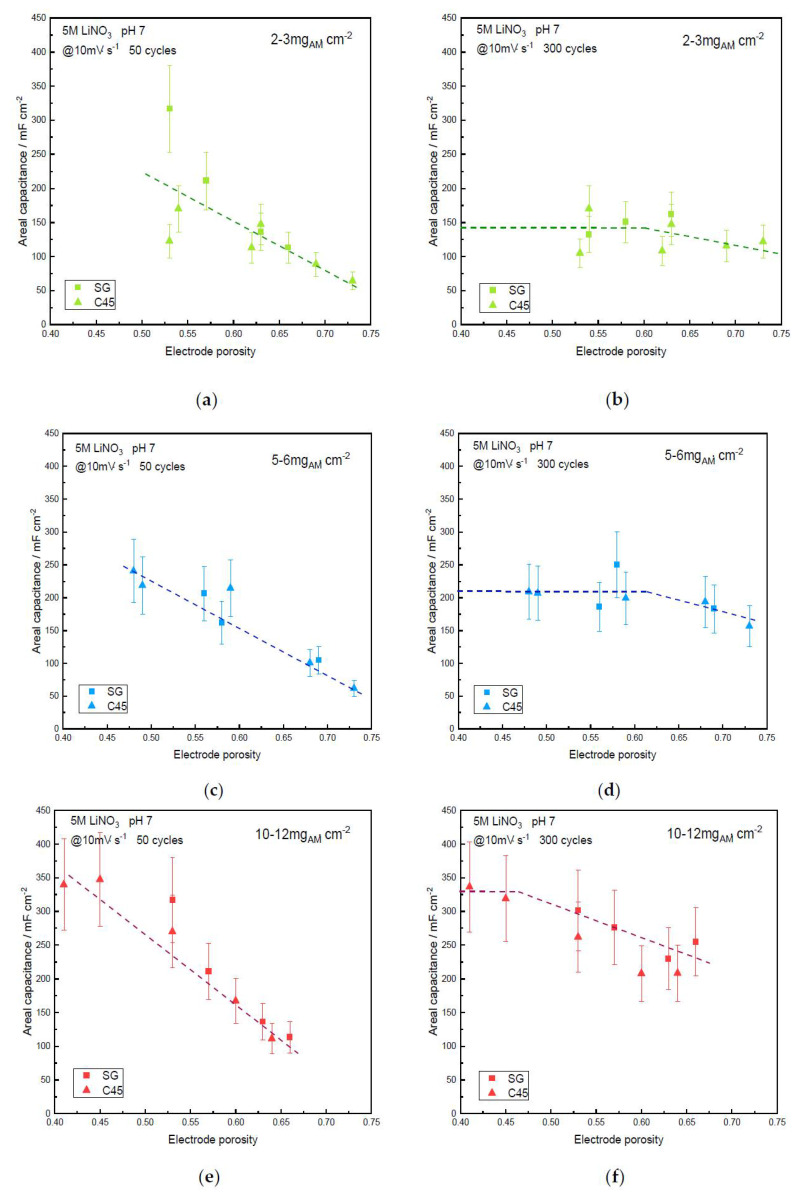
Areal capacitance vs. electrode porosity of the α-MnO_2_ symmetric supercapacitor device using SG or C45 carbon black additive, after cycling in 5M LiNO_3_ at 10 mV·s^−1^: (**a**) Low-mass loading, 50 cycles; (**b**) Low-mass loading, 300 cycles; (**c**) Medium-mass loading, 50 cycles; (**d**) Medium-mass loading, 300 cycles; (**e**) Large-mass loading, 50 cycles; (**f**) Large-mass loading, 300 cycles. Dashed lines are guidelines for the eyes.

Electrochemical results about the two-electrode symmetric device using the α-MnO_2_ electrodes are reported in Figure 4 and Figure 5 for 10 mV·s^−1^ scan rate showing the specific (Figure 4) and areal (Figure 5) capacitances versus the electrode porosity (for the two carbon additives; namely, SG and C45 carbon blacks), for the three loadings, low (2–3 mg·cm^−2^), medium (5–6 mg·cm^−2^), large (10–12 mg·cm^−2^), and after 50 and 300 cycles. Specific device capacitances range from 5 F·g^−1^ and 25 F·g^−1^ and areal capacitances from 50 to 350 mF·cm^−2^ depending on the experimental conditions.

#### 3.4.1. Effect of the Porosity and Mass Loading

First, the nature of the carbon black, SG or C45, does not seem to influence the specific or areal capacitances. It can be noted that the highest porosity (~75%) is only 6.5% less than the tapped density of the α-MnO_2_ powder (81.5%). Thus, the casting onto the current collector only results in a very small decrease in porosity. This slight decrease can be assigned to the presence of the tiny particles of carbon black additive, SG or C45, which fills some gaps between MnO_2_ particles during the tape-casting process.

An increase of the specific and areal capacitances after 50 cycles is observed when the porosity decreases, whatever the mass loading. This can be explained by the steady wetting of the electrodes by the electrolyte upon cycling the cell. This demonstrates that there is some hindrance to the access of the liquid electrolyte to the electrode surfaces. Interestingly, the capacitance values seem to stabilize for porosities down to 55%, which suggests a faster wetting of the dense electrodes. Indeed, the tortuosity of the more porous electrodes prevents fast wetting. This highlights one of the advantages of calendering the electrodes and this is probably one of the reasons why this process is used for the fabrication of commercial devices. Keeping the electrode porosity above 55% leads to a lower wetting and this must be taken into account when determining capacitance values. Even after 300 cycles, only the devices with electrodes loaded with the lower mass loading and those with low electrode porosities (<60% for 5–6 mg_AM_·cm^−2^ and <50% for 10–12 mg_AM_·cm^−2^) succeed to reach a constant value of the capacitance. This pointed out the importance of the wetting process which is directly related to the electrode loading and porosity. Subsequently, if the electrode porosity is too high and/or the mass loading is too high, the maximum capacitance of the device is not reached even after 300 cycles. Unfortunately, most of the literature in the field does not wait such a long time to report on device capacitance. Indeed, there are other means to fasten the wetting of the electrodes, such as impregnation under vacuum, but again, such methods are scarcely reported in the experimental sections of related papers. Moreover, prior to any wetting, low- (<50%) and high- (>70%) porosity electrodes (10–12 mg_AM_·cm^−2^) exhibited exactly the same water contact angle (135 ± 5° using aqueous 5M LiNO_3_), which indicates a hydrophobic behavior. However, it seems that the electrolyte most easily penetrated the low-porosity one when the devices were assembled, probably due to a denser network of hydrophilic MnO_2_ particles.

The maximum specific capacitances reach 25 F·g^−1^ (measured at 10 mV·s^−1^) for the lower-mass loading (2–3 mg·cm^−2^), showing a slight decrease when the mass loading increases. After 300 cycles, the gravimetric capacitance of the films with the lowest porosity is 24 F·g^−1^ for 2–3 mg_AM_·cm^−2^, 22 F·g^−1^ for 5–6 mg_AM_·cm^−2^ and reaches 20 F·g^−1^ for 10–12 mg_AM_·cm^−2^. For such loading, it must be noted that a capacitance of 28 F·g^−1^ at 10 mV·s^−1^ was expected according to the three-electrode cell measurement (Figure 3a). The discrepancy between the capacitance value measured in a three-electrode cell in a beaker filled with electrolyte and magnetic stirring promotes electrode wetting and is poorly dependent on the geometry of the cell, while for the device, electrodes are cut, assembled with a separator between them and pressed by metallic clamps, making the whole device strongly dependent upon the geometry used: alignment of face-to-face for the electrode, poor initial wetting, etc. Thus, it seems that capacitance values extrapolated from device measurements are underestimated compared to the electrochemical investigation of a single electrode.

It must also be noted that the related energy density shows a 50% decrease when the mass loading is increased from 2 to 3 mg_AM_·cm^−2^ up to 10 to 12 mg_AM_·cm^−2^ for ~ 75% porosity. This trend is attenuated when the porosity is decreased by using the calendering process. Indeed, the discrepancy between low- and high-mass loadings is much lower for electrodes with only 50% porosity, with a 20% difference in the capacitance of the full device. This is a very interesting illustration of the influence of the mass loading and the calendering step. Obviously, most of the values reported in the literature are provided with low-mass loading and high porosity, which leads to an overestimation of the capacitance and consequently of the energy density of the devices that can reach +50% compared to the values measured for the electrodes with high-mass loading. It can be noted that even the medium-mass loading does not enable the as-coated electrode to reach the capacitance of those with 2–3 mg_AM_·cm^−2^. The usefulness of the calendering process is emphasized since it significantly helps to decrease this discrepancy between low- and high-mass loading when porosity is decreased down to 50%. It can be noted that unlike batteries, for which porosities as low as 20% have been measured [44], electrochemical capacitors require a certain level of porosity to mitigate ionic conductivity and electronic conductivity.

The areal capacitances of the different electrodes are presented in Figure 5. As expected, the delay in obtaining a full wetting of the electrodes is also observed, and the electrodes with the lowest porosity are those faster wetted. The plot also evidenced a larger scattering of capacitance values when low-mass loading is used (Figure 5a).

The areal capacitance stabilizes after 300 cycles, although this is not totally the case for high-mass-loading electrodes with large porosity. However, the main discrepancy between the three mass loadings can be highlighted from the areal capacitance, which is the performance of importance for fabricating commercial devices. While for low-mass loading the areal capacitance of the device is limited to 150 mF.cm^−2^, this value increases to 225 mF·cm^−2^ for medium-mass loading and up to 350 mF·cm^−2^ for large-mass loading. For the higher-mass loading, the maximum areal capacitance is measured for the lowest porosity (<50%). Such high-mass loading is close to the values expected for real-life electrodes. Thus, when trying to give a meaningful idea of a device’s capacitance, authors should report capacitance values on 10–12 mg_AM_·cm^−2^ loaded electrodes which have been laminated down to 50% porosity.

**Figure 6:**
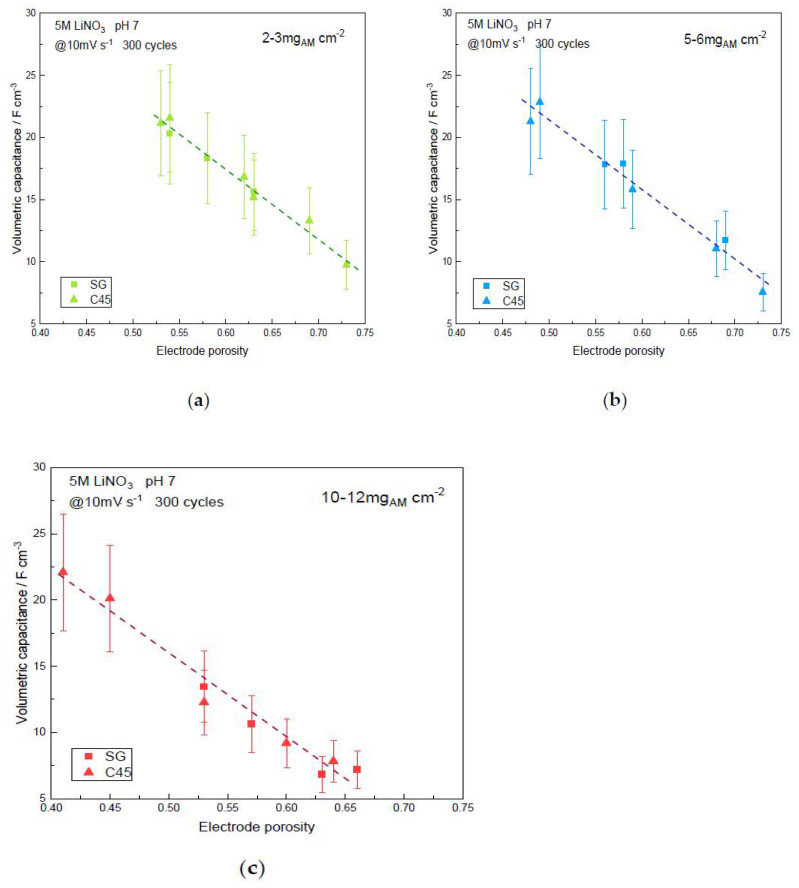
Volumetric capacitance vs. electrode porosity of the α-MnO_2_ symmetric supercapacitor device using SG or C45 carbon black additive, after 300 cycles in 5M LiNO_3_ at 10 mV·s^−1^: (**a**) Low-mass loading; (**b**) Medium-mass loading; (**c**) Large-mass loading. Dashed lines are guidelines for the eyes.

From Figure 6 it is obvious that the volumetric capacitance strongly depends upon the electrode porosity, the highest values being achieved for the lowest porosity, thus pointing out again the role of the calendering process. The slope is almost the same for the three mass loadings. Extrapolation of the plots to 40% porosity illustrates a volumetric capacitance of 30 F·cm^−3^ for the low- and medium-mass loadings while the high-mass loading seems to be limited to 23 F·cm^−3^. Again, this is a 30% decrease in capacitance, which in turn leads to a 30% decrease in energy density for the device with the largest mass loading.

Again, the role of the calendering process is highlighted when focusing on the volumetric capacitance: there is a linear relationship between porosity and capacitance. Commercial devices are fabricated with spirally wounded electrodes and it does not sound realistic to assemble such devices with electrodes presenting high porosity which are in the range of 5–10 F·cm^−3^. Meanwhile, electrodes prepared with a calendering step and laminated down 50% porosity exhibit two to four times higher volumetric capacitance.

#### 3.4.2. Electrochemical Impedance Spectroscopy

**Figure 7:**
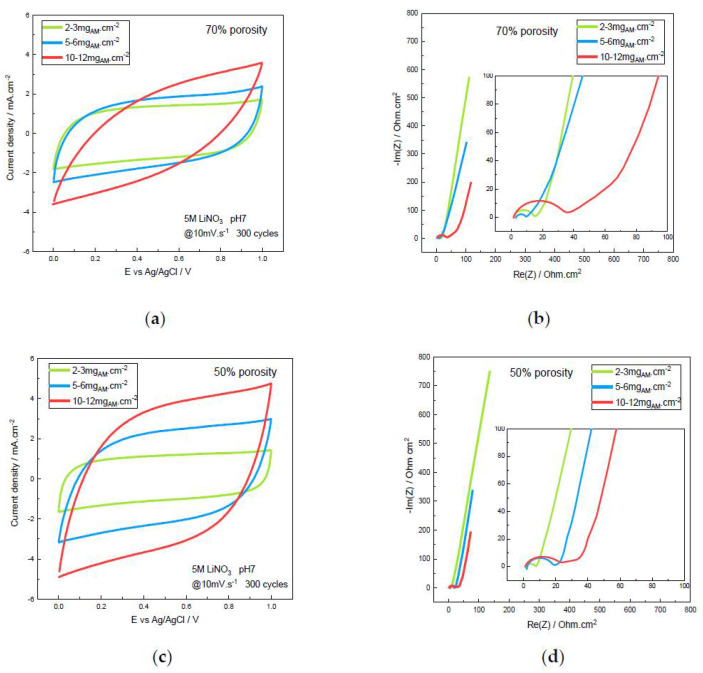
Cyclic voltammetry and electrochemical impedance spectroscopy of the α-MnO_2_ symmetric supercapacitor device after 300 cycles in 5M LiNO_3_ at 10 mV·s^−1^ for low-, medium- and large- mass loading at two different porosities: (**a**) 70% porosity, CV; (**b**) 70% porosity, Nyquist diagram (200 kHz–2 mHz at E_OC_, 20 mV potential amplitude); (**c**) 50% porosity, CV; (**d**) 50% porosity, Nyquist diagram (200 kHz–2 mHz at E_OC_, 20 mV potential amplitude) (inserts: magnification of the Nyquist diagrams).

These results are confirmed in Figure 7 by Electrochemical Impedance Spectroscopy (EIS) investigated after 300 cycles at 10 mV·s^−1^ with the devices at ~50% and ~70% porosities and different mass loadings. The internal resistance, including the ohmic resistance, the charge transfer resistance, double-layer capacitance and the Warburg resistance systematically increases slightly after 300 cycles compared to the initial state not shown here. Obviously, Figure 7b,d show that the high-mass loading especially induces a lower internal resistance at 50% porosity with the calendering treatment compared to the 70% porosity, as shown also by the more rectangular CV observed in Figure 7c at 50% of porosity compared to the 70% porosity in Figure 7a. All the Nyquist plots exhibit an almost vertical impedance at low frequencies, characteristic of a capacitive behavior, strictly parallel for the three mass loadings at 50% porosity. This part of the Nyquist diagram determines the areal capacitance from the maximal Z” areal imaginary impedance value at the lowest frequency (f = 2 mHz), given by the equation [9]:

C = 1/(2πf(−Z”))(3)

An increase in areal capacitance is confirmed from EIS measurements for both porosities when the mass loading increases (Table 1).

The swelling at higher frequencies (insert in Figure 7b,d) shows the R_ohmic_ ohmic resistance at the beginning of the Nyquist plot, which is the ionic resistance of the electrolyte supported by the separator, a depressed semi-circular feature of the R_ct_ charge transfer resistance and C_dl_ double-layer capacitance, usually obtained at the electrode/electrolyte interface for porous materials, and a Z_D_ diffusion impedance, close to a Warburg impedance, assigned to the diffusion and mass-transport control at intermediate frequencies, depending on the porosity and thickness of the electrodes. EIS experiments are fitted using resistors (R) and constant phase elements (CPE) according to series-equivalent circuits such as the Randles circuit or an open-uniform distributed RCPE transmission line model. The resulting values are given in Table 1. 

The ohmic resistance, the ionic resistance of the electrolyte, does not reveal any change with porosity or mass loading. The R_ct_ charge transfer resistance and the Z_D_ diffusion impedance decrease when the porosity and then thickness decreases, as well as when the mass loading decreases. This pseudocapacitive behavior reveals a charge transfer and mass transport slower in the porous and thicker electrodes which were depicted from the capacitance values reported from CVs and GCD measurements. Decreasing the porosity by calendering is advantageous for the electrochemical behavior at the surface electrode especially for high-mass loadings, reducing the charge transfer resistance and increasing the double-layer capacitance. A similar evolution observed with the diffusion impedance, namely, an increase when porosity and mass loading increase, is significant of the diffusion and mass transport in the electrode, making easier and faster the diffusion in the dense material, especially for the high-mass-loading electrodes.

**Figure 8:**
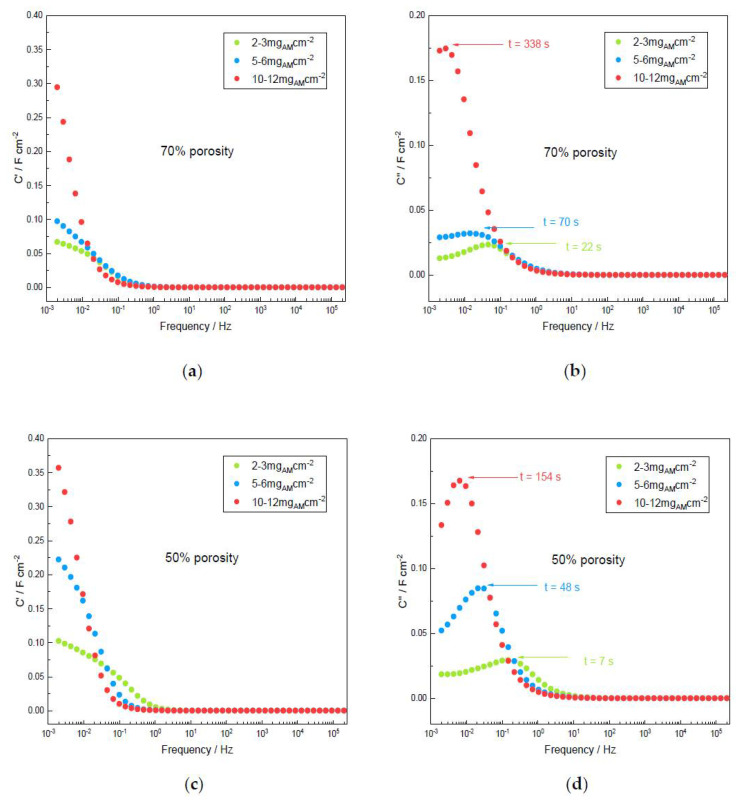

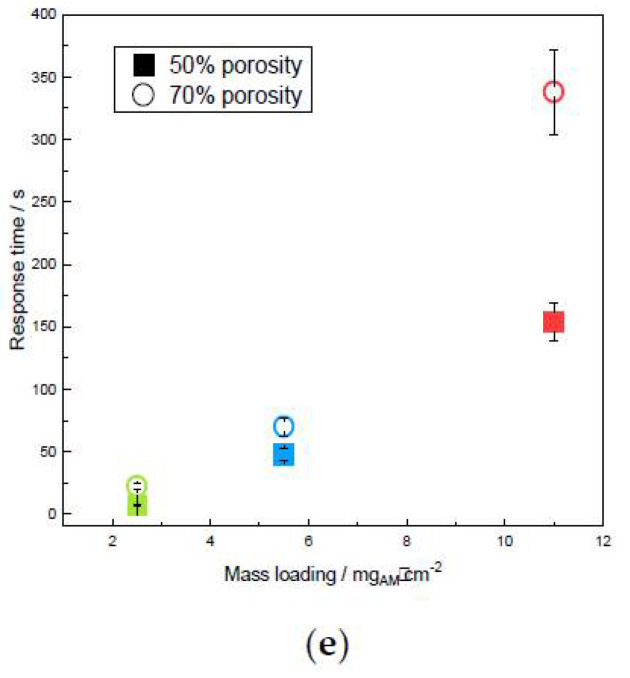
Bode plots of C’ and C’’ capacitances vs. frequency (Ref [32] for the calculation method) and response time of α-MnO_2_ symmetric device vs. the mass loading for both porosities: (**a**) C’ for 70% porosity; (**b**) C” for 70% porosity; (**c**) C’ for 50% porosity; (**d**) C” for 50% porosity; (**e**) Response time vs. mass loading for 50% and 70% porosity.

The Bode plot drawing the real and imaginary capacitances of C’ and C” vs. frequency is represented in Figure 8. It allows for the determination of the rate capability of the MnO_2_ electrode by determining the time constant related to the pseudocapacitive behavior of the electrodes implemented in a full device as a function of the mass loading and porosity. Large mass loadings lead to a higher time constant, namely, slower reaction kinetics, mass transport and diffusion, as previously described for a carbon–carbon supercapacitor [32]. Whereas decreasing the porosity speeds up the electrochemical mechanisms and decreases the time constant in a considerable way for high-mass loadings, low and medium ones lead to similar time constants. These plots also reveal that the time response determination conducts, suggesting that a high-mass loading device seems to work as a power battery whereas electrode calendaring, which allows for decreasing of the time response, leads to an electrical behavior closer to that expected for a supercapacitor. This situation is commonly found in a device made of pseudocapacitive materials for which the kinetic of charge is much less than for double-layer capacitors using activated carbon electrodes. Unfortunately, the time constant of such a device is rarely extracted from EIS measurements even if it is often larger than several tens of seconds. Thus, full devices implemented with pseudocapacitive electrodes are less powerful devices than commercial ECs although the energy density can be higher thanks to larger capacitance values. Our MnO_2_-based device presented herein can be efficiently charged/discharged in a few minutes but not in a few seconds. This is also an intrinsic drawback of MnO_2_ as active material since lab-scale prototypes use activated carbon as active material, either in an aqueous or organic electrolyte. Similar preparation techniques for symmetrical devices (Kuraray YP50F carbon based electrodes) lead to a response time of 0.36 s [45] in aqueous 5M LiNO_3_ electrolyte, and 9 s in propylene carbonate-based electrolyte [7].

It can also be noted that performing EIS on a single electrode in a three-electrode cell is helpless to access the time constant of a future device, even if many authors are extracting kinetic data from a single electrode to report on energy and power densities in a Ragone plot.

## 4. Conclusions

The roles of the electrodes’ mass loading and calendering were investigated in order to identify their influence on the performance of cryptomelane-type α-MnO_2_ symmetric devices. The electrochemical performance of such devices was improved in neutral aqueous electrolytes after applying successive calendering steps to vary the porosity from the as-prepared coating (~70% porosity) to less than 50% porosity. Specifically, areal and volumetric capacitances increase when the porosity decreases, whatever the mass loading. This suggests a faster wetting of a dense electrode by the electrolyte while the tortuosity of a porous electrode seems to prevent fast wetting. Only devices with low-mass loading and those with low porosities succeed to reach stable capacitances values after 300 charge/discharge cycles. 

A significant discrepancy exists between low- and high-mass loading, leading to an expected increase of the related energy density when the mass loading decreases, especially for porous electrodes (70%). Thus, using low-mass loading and high porosity induce an overestimation of more than 50% of the gravimetric capacitance and the specific energy density of the device. The calendering process significantly decreases this discrepancy between the low and the more realistic mass loading (10–12 mg_AM_·cm^−2^). Nevertheless, high-mass loading with the lowest porosity leads to a maximum areal capacitance compared to all the other designs. Volumetric capacitance too strongly depends upon the electrode porosity, without mass-loading dependence, giving still the highest value for the lowest porosity. However, in this last case, the volume of current collectors should also be included in the calculations, which will drastically lower the benefit of low-mass loading since the volume of the electrode will be less than the volume of metallic current collectors in such a case. Again, a realistic evaluation of the device performance goes through the fabrication of calendered electrodes with high-mass loading.

All these findings allow us to provide the following advice that can serve as guidelines for the evaluation of electrode and device performance for supercapacitors. Most of these guidelines can also be translated to the battery field:

(1) The mass loading of an electrode drastically influences the reported values, either gravimetric, areal or volumetric. Moreover, the capacitance for real-life mass loading (10–12 mg_AM_·cm^–2^) is not stabilized even after 300 cycles in the aqueous-based electrolyte. Thus, long-term pre-cycling of the electrodes must be achieved (or an alternative wetting method must be used) prior to reporting on the performance. This pre-cycling step must be clearly detailed in the experimental section of the papers.
(2) Lower mass loadings (<5–6 mg_AM_·cm^−2^) systematically lead to an overestimation of capacitance values. Although it is sometimes not possible to prepare electrodes with high-mass loadings, it is mandatory to report on the mass loading achieved for the measured electrodes in the experimental section. Just providing the electrode composition is definitely not enough to give the readers a realistic idea of their performance.
(3) The porosity of the electrode is a key parameter that drastically influences the performance of electrodes and devices. It is not expected for all the research groups to be equipped with the calendering process, but it is mandatory to pertinently report on the porosity of the electrodes. Only a few groups are aware of such an influence of electrode porosity which should be ideally decreased down to 50%. Such a parameter is easy to determine by measuring the average thickness of the electrode and its loading. The corresponding volumetric loading (in g_electrode_·cm^−3^) must be compared to the volume that should fill a composite electrode of the same mass if all its components were densely packed onto the current collector.
(4) Lastly, investigating a single electrode in a three-electrode cell is a mandatory step before assembling any device, but it does not enable the extrapolation to device performance, neither in terms of capacitance nor time constant.

## Figures and Tables

**Table 1:** Data values from the EIS experiments.

Porosity	Mass Loading	R_ohmic_	C_dl_	R_ct_	Z_D_	−Z”	C
%	mg_AM_·cm^−2^	ohm·cm^2^	μF·cm^−2^	ohm·cm^2^			mF·cm^−^^2^
70%	Low	1.18	1.50	13.76	8.61	574	139
	Medium	2.30	1.59	6.95	14.39	344	231
	Large	1.27	4.66	32.47	39.56	201	396
50%	Low	1.14	3.25	6.10	3.47	752	106
	Medium	1.71	4.37	15.57	4.11	339	235
	Large	0.68	10.65	22.29	10.33	196	406

## Data Availability

The data presented in this study are available on request from the corresponding author. The raw data are not publicly available due to the use of commercial material.
